# Lack of Evidence Supporting a Significant Benefit of Pre-Transplant Consolidation Therapy in AML CR2 Patients Undergoing Allogeneic Hematopoietic Stem Cell Transplantation

**DOI:** 10.3390/cancers17081364

**Published:** 2025-04-19

**Authors:** Meng Lv, Ting Huang, Xiao-Dong Mo, Yu-Qian Sun, Ying-Jun Chang, Lan-Ping Xu, Xiao-Hui Zhang, Xiao-Jun Huang, Yu Wang

**Affiliations:** 1 Peking University People’s Hospital, Peking University Institute of Hematology, National Clinical Research Center for Hematologic Disease, Beijing Key Laboratory of Cell and Gene Therapy for Hematologic Malignancies, Beijing 100044, China; drlvmeng@bjmu.edu.cn (M.L.);; 2 Peking-Tsinghua Center for Life Sciences, Beijing 100084, China; 3 State Key Laboratory of Natural and Biomimetic Drugs, Peking University, Beijing 100083, China

**Keywords:** allo-HSCT, AML, second complete remission, consolidation

## Abstract

**Background**: Allogeneic hematopoietic stem cell transplantation (HSCT) is a well-established curative treatment option for acute myeloid leukemia (AML) in second complete remission (CR2). However, whether the addition of consolidation chemotherapy after achieving CR2 can improve transplant outcomes remains controversial. **Methods**: In this single-center retrospective study, we analyzed consecutive AML patients who underwent their first HSCT in CR2 at our institution between January 2015 and December 2019. **Results**: For the consolidation (*n* = 72) and no consolidation groups (*n* = 63), the 5-year cumulative incidence of relapse (CIR) was (17.6% vs. 19.9%; *p* = 0.54), the 5-year non-relapse mortality rate (NRM) was (9.7% vs. 17.5%; *p* = 0.20), the 5-year leukemia-free survival (LFS) was (72.7% vs. 62.7%; *p* = 0.15), and the 5-year overall survival (OS) was (81.9% vs. 68.3%; *p* = 0.08). Additional consolidation therapy to achieve negative measurable residual disease (MRD) did not result in significantly improved outcomes compared to immediate HSCT in MRD positive status, with similar LFS (76.9% vs. 67.0%, *p* = 0.2) and OS (88.3% vs. 75.0%, *p* = 0.14). Multivariable analysis indicated that consolidation chemotherapy did not significantly affect CIR, NRM, LFS, or OS. **Conclusions**: Our findings suggest no significant differences in clinical outcomes between the groups, indicating that AML patients in CR2 might proceed to HSCT without delay.

## Simple Summary

Stem cell transplantation can offer a potential cure for people with acute myeloid leukemia who have achieved a second complete remission. However, it is uncertain whether giving additional chemotherapy before the transplant improves long-term survival. In this study, we compare outcomes between those who received extra chemotherapy before transplant and those who did not. We found no significant differences in survival, risk of relapse, or non-relapse mortality between the two groups. Even for patients who still had measurable residual diseases before transplant, the additional chemotherapy did not lead to better results. These findings suggest that going directly to transplant after achieving remission without delaying may be just as effective.

## 1. Introduction

Although approximately 60–80% of de novo acute myeloid leukemia (AML) patients achieve complete remission (CR) by induction chemotherapy, the risk of recurrence among these patients remains high due to the high degree of clinical heterogeneity of AML [1]. Relapsed AML following induction chemotherapy remains a highly lethal disease [2], with a median survival of ~6 months after first recurrence in such patients [3,4,5]. AML patients at their first CR (CR1) who relapse after conventional chemotherapy have limited treatment options and allogeneic hematopoietic stem cell transplantation (HSCT) is still the only reliable curative modality [6,7,8]. In these patients, re-induction chemotherapy is often given to achieve a CR2 to allow for HSCT [9]. Not surprisingly, patients who underwent HSCT in CR2 had worse clinical outcomes than those who underwent HSCT at CR1 [6,10,11].

In the non-transplant setting, it is an indisputable fact that consolidation chemotherapy is effective. Repeated cycles of consolidation chemotherapy have been demonstrated to improve clinical prognosis [12,13]. Thus, consolidation chemotherapy is considered the standard of care for the cytogenetically favorable AML group after remission [14,15]. In the context of HSCT, three large retrospective studies did not show the beneficial effects of pre-transplant consolidation chemotherapy [16,17,18].

Recently, results from a randomized, open-label, phase III, non-inferiority trial indicated that remission induction followed by HSCT was not superior to immediate HSCT for patients with relapsed AML or AML with poor responsiveness. The study recommended that salvage chemotherapy may be omitted and immediate HSCT may be offered if a donor is available for such patients [19]. Yet, some independent studies have shown opposite conclusions [20,21,22]. In a retrospective analysis, Liu J et al. found that recipients of MSDT (matched sibling donor transplantation) in their CR1 who received three or more pre-transplant consolidation regimens had significantly higher overall survival (OS) and significantly lower cumulative incidence of relapse (CIR) compared to those who received two or fewer courses of consolidation therapy [20]. The question of whether AML transplant recipients in CR should receive consolidation chemotherapy before undergoing HSCT remains unresolved.

However, most studies have concentrated on the value of pre-transplant consolidation chemotherapy for AML patients in their CR1. Patients in CR2 typically have a higher leukemic tumor burden compared to those in CR1. While the potential therapeutic value of consolidation chemotherapy for AML patients in CR2 undergoing HSCT is of interest, there is a scarcity of clinical data addressing this issue. Therefore, this retrospective study evaluated the impact of pre-transplant consolidation chemotherapy on post-transplant outcomes in CR2 AML patients receiving HSCT.

## 2. Methods

Inclusion criteria: Patients diagnosed with AML who underwent their first HSCT while in their CR2 at our center between January 2015 and December 2019. The study was approved by the Ethics Committee of Peking University People’s Hospital and complied with the Declaration of Helsinki.

### 2.1. Transplantation Protocol

Haplo-HSCT, HLA-matched unrelated donor transplantation (MUDT), and MSDT were performed exactly as described in protocols previously reported by our group [20,23,24]. The conditioning regimen of MSDT was as follows: hydroxycarbamide (80 mg/Kg day −10 d), cytarabine (Ara-C) (2 g/m^2^/day −9 d), busulfan (Bu) (3.2 mg/kg/day i.v from days −8 to −6), cyclophosphamide (CTX) (1.8 g/m^2^/day i.v from days −5 to −4), simustine (250 mg/m^2^/days −3 d). The conditioning regimen of MUDT was as follows: Ara-C (2 g/m^2^/day i.v from days −10 to −9), Bu (3.2 mg/kg/day i.v from days −8 to −6), CTX (1.8 g/m^2^/day i.v from days −5 to −4), simustine (250 mg/m^2^/day −3 d), rabbit thymoglobulin (ATG) (2.5 mg/kg/day i.v from days −5 to −2). The conditioning regimen of haplo-HSCT was as follows: Ara-C (4 g/m^2^/day i.v from days −10 to −9), Bu (3.2 mg/kg/day i.v from days −8 to −6), CTX (1.8 g/m^2^/day i.v from days −5 to −4), simustine (250 mg/m^2^/day −3 d), ATG (2.5 mg/kg/day i.v from days −5 to −2) [25,26].

### 2.2. Graft-Versus-Host Disease (GVHD) Prophylaxis

GVHD prophylaxis regimens were as previously described and briefly reviewed below [27]. Patients who underwent MSDT received a cyclosporine A (CsA) + short-term methotrexate (MTX)-based GVHD prophylaxis regimen. Patients who underwent MUDT or haplo-HSCT received a CsA + short-term MTX + anti-thymocyte globulin (ATG)-based GVHD prophylaxis regimen.

### 2.3. Definitions

The 2022 European LeukemiaNet (ELN) recommendations for AML were used for the risk stratification of patients [28]. Patients with a prior history of solid tumors were excluded. Acute GvHD (aGvHD) was staged according to the Keystone Criteria established in 1994 [29], and chronic GVHD (cGvHD) was graded according to the National Institutes of Health consensus criteria [30]. Neutrophil engraftment was defined as an absolute neutrophil count (ANC) ≥ 0.5 × 10^9^/L for 3 consecutive days. Platelet engraftment was defined as a platelet count > 20 × 10^9^/L for 7 consecutive days without platelet transfusion support. Minimal residual disease (MRD) was assessed by multiparameter flow cytometry (MFC) with a sensitivity of at least 0.01%. Positive MRD was defined as any disease detectable by MFC [31,32,33]. Time to the clinical endpoint event was measured from the date of HSCT. Relapse was defined as hematologic recurrence of leukemia. Leukemia-free survival (LFS) was defined as survival without leukemia relapse. OS was defined as death from any cause taken from the date of HSCT. Non-relapse mortality (NRM) was defined as death without leukemia relapse after HSCT.

### 2.4. Statistical Analysis

An independent samples t-test was used for normally distributed continuous variables, and the results were expressed as mean and standard deviation (SD). The Mann-Whitney U test was used for non-normally distributed continuous variables, and the results were expressed as the median and interquartile range (IQR). The chi-square test and Fisher’s exact test were used for categorical variables. The probabilities of LFS and OS were calculated using the Kaplan–Meier method [34]. Cumulative incidences of CIR, NRM, and GVHD were calculated using the competing risks method [35]. The log-rank test and Fine–Gray test were performed for univariable analysis. Multivariable analysis was conducted using the Cox proportional hazards model and competing risk models to calculate hazard ratios (HRs) and 95% confidence intervals (CIs). We fitted the proportional hazards model, after confirming that the proportional hazards assumption was met. NRM and relapse were competing risks for each other. In addition, death from any cause was a competing risk for GVHD. All statistical tests were two-sided and *p* < 0.05 was considered statistically significant. R 4.2.2 (R Foundation for Statistical Computing, Vienna, Austria) was performed for analyses and plots.

## 3. Result

### 3.1. Patients’ Characteristics and Outcomes

This study included 135 AML patients who underwent HSCT in CR2, comprising 72 patients who received pre-transplant consolidation chemotherapy and 63 who did not (Table 1). Among the 72 patients receiving consolidation therapy, 50 (69.4%) underwent the treatment to eliminate pre-transplant MRD, with 80% achieving MRD negativity before HSCT. Meanwhile, 15 patients (20.8%) experienced delays in HSCT due to uncontrolled infections or other comorbidities, and 7 patients (9.7%) faced delays related to donor availability, such as the search for unrelated donors or the reduction in donor-specific antibodies (DSAs) (Supplement Table S1). MRD negativity was significantly more common in the consolidation group compared to the non-consolidation group (79.2% vs. 55.6%, *p* = 0.003). Of the 39 HSCT patients with positive pre-transplant MRD, 28 received no consolidation therapy, 10 received one course, and 2 received multiple courses. However, there were no statistically significant differences in MRD negativity between patients receiving one versus multiple rounds of consolidation therapy (74.0% vs. 90.9%, *p* = 0.1719) (Supplement Table S2, Supplement Figure S1).

The median (IQR) follow-up for the entire cohort was 55.0 months (34.9–72.6 months). The 5-year CIR, NRM, LFS, and OS were 18.7%, 13.3%, 68.0%, and 75.6%, respectively. For the entire cohort, the median IQR time to neutrophil engraftment and platelet engraftment was 13 days (12–15 days) and 17.5 days (13–21.3 days), respectively. Six patients failed to achieve platelet engraftment within 100 days post-HSCT, including one patient who died 5.4 months post-HSCT without achieving platelet engraftment. The cumulative incidence of grade II–IV aGvHD at day 100 was 18.5%, and the cumulative incidence of cGvHD was 46.4%.

### 3.2. Impact of Pre-Transplant Consolidation Therapy on HSCT

**Figure 1:**
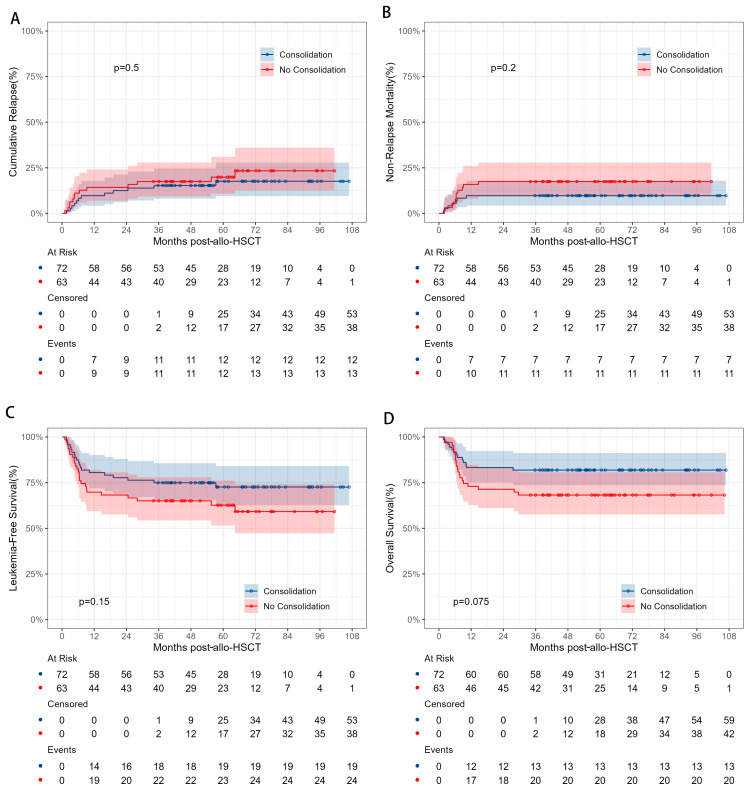
Comparisons of CIR (**A**), NRM (**B**), LFS (**C**), and OS (**D**) between AML patients in CR2 with and without pre-transplant consolidation chemotherapy before HSCT.

The overall prognosis of CR2 AML patients with and without pre-transplant consolidation chemotherapy was comparable. For the consolidation and no-consolidation groups, the 5-year CIR was 17.6% vs. 19.9% (*p* = 0.54), the 5-year NRM was 9.7% vs. 17.5% (*p* = 0.20), the 5-year LFS was 72.7% vs. 62.7% (*p* = 0.15), and the 5-year OS was 81.9% vs. 68.3% (*p* = 0.08). Similarly, the 100-day cumulative incidence of grade 2–4 aGvHD was 18.1% vs. 19.0% (*p* = 0.84), and the 5-year cumulative incidence of cGvHD was 52.2% vs. 39.7% (*p* = 0.29) (Table 2 and Figure 1).

Univariable analyses showed that age at transplantation greater than or equal to 30 years or ≥2 courses of induction chemotherapy from relapse to CR2 had an unfavorable impact on OS and LFS, and that the risk of NRM was higher in patients aged 30 years or older. Patients with pre-transplant MRD positive had a higher risk of relapse than those with MRD negative (Supplement Table S3).

Next, the six variables with the lowest *p* values in the univariable analyses were included in the multivariable analyses. The results indicated that pre-transplant MRD positivity remained a strong and independent prognostic factor for relapse. A recipient age of ≥30 years was consistently associated with an increased risk of NRM. Additionally, pre-transplant consolidation chemotherapy showed no significant impact on transplant outcomes in the HSCT setting, as confirmed by both univariable and multivariable models. Furthermore, none of the covariates analyzed demonstrated a significant effect on the incidence of GVHD following HSCT in this study (Table 3).

### 3.3. Effect of the Number of Pre-Transplant Consolidation Therapy Courses on HSCT Outcomes

**Figure 2:**
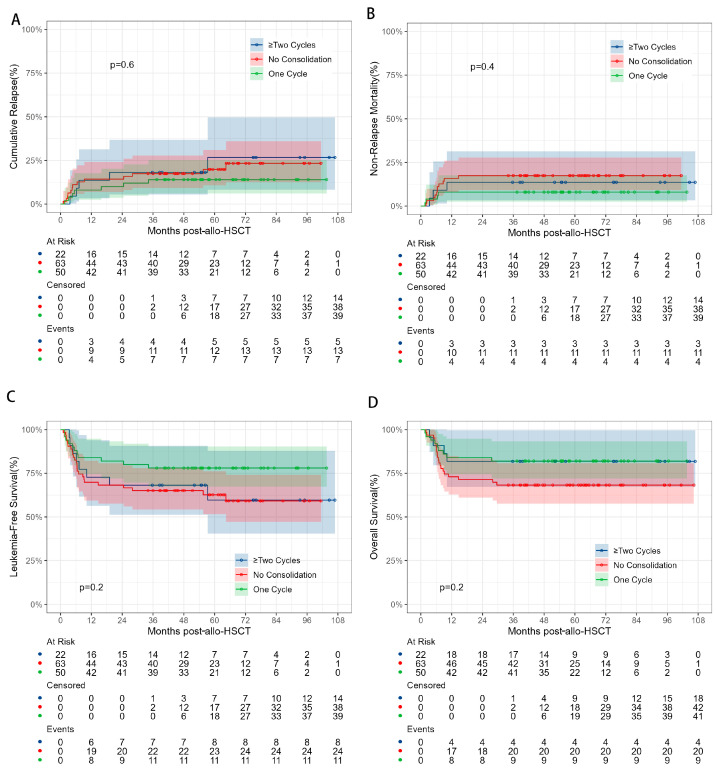
Comparisons of CIR (**A**), NRM (**B**), LFS (**C**), and OS (**D**) between AML patients in CR2 with different courses of pre-transplant consolidation chemotherapy before HSCT.

To evaluate the impact of the number of pre-transplant consolidation therapies on the clinical outcomes of CR2 AML patients after HSCT, the patients were divided into three groups based on the number of consolidations: no consolidation therapy group (*n* = 63), 1-consolidation therapy group (*n* = 50), and ≥2-consolidation therapy group (*n* = 22) (Supplement Tables S1 and S4). Notably, the 5-year CIR for the no-consolidation, 1-consolidation, and ≥2-consolidation therapy groups was 19.9%, 14%, and 26.7%, respectively (*p* = 0.5855). While the differences were not statistically significant, the 1-consolidation therapy group appeared to show a trend toward reducing the risk of relapse, whereas the ≥2-consolidation therapy group exhibited worse clinical outcomes compared to the no-consolidation therapy group (Figure 2).

### 3.4. Impact of Pre-Transplant MRD Status on HSCT Outcomes

**Figure 3:**
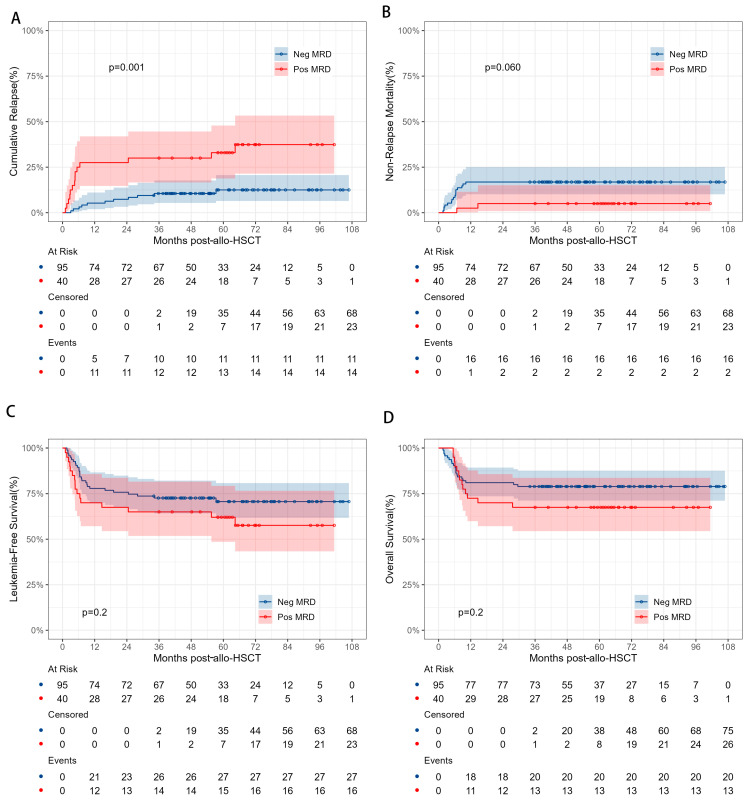
Comparisons of CIR (**A**), NRM (**B**), LFS (**C**), and OS (**D**) between AML patients in CR2 with different MRD statuses before HSCT.

To evaluate the impact of pre-transplant MRD on HSCT outcomes, patients were stratified into a pre-transplant MRD+ group (*n* = 40) and a pre-transplant MRD− group (*n* = 95). The pre-transplant MRD+ and MRD− groups showed similar probabilities of 5-year OS (68.3% vs. 81.9%, *p* = 0.2), 5-year LFS (62.7% vs. 72.7%, *p* = 0.2), and 5-year NRM (5.0% vs. 16.8%, = 0.06), but the pre-transplant MRD+ group had a significantly higher 5-year CIR (33.0% vs. 12.5%, *p* = 0.001) (Figure 3).

**Figure 4:**
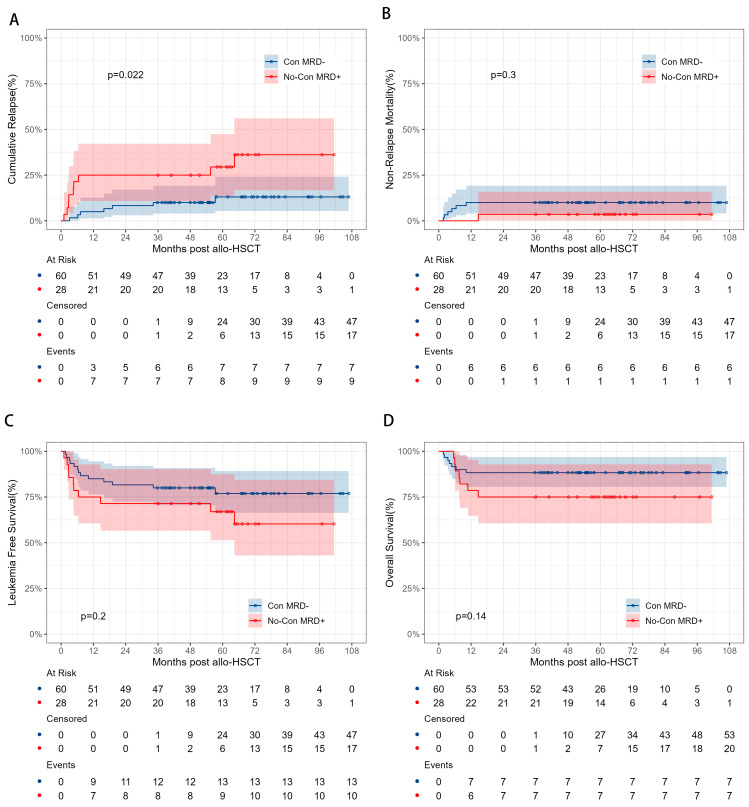
Comparisons of CIR (**A**), NRM (**B**), LFS (**C**), and OS (**D**) between AML patients in CR2 with immediate HSCT in MRD+ or MRD− through additional consolidation therapy.

To assess whether achieving MRD− status through additional consolidations (*n* = 60) yields better outcomes compared to immediate HSCT in MRD+ status (*n* = 28), the results showed a higher 5-year CIR in the MRD+ group (29.5% vs. 13.1%, *p* = 0.022). However, no significant differences were observed in 5-year OS (88.3% vs. 75.0%, *p* = 0.14), 5-year LFS (76.9% vs. 67.0%, *p* = 0.2), or 5-year NRM (10.0% vs. 3.6%, *p* = 0.3) (Figure 4).

In patients with pre-transplant MRD-negative CR2 AML, one course of consolidation appeared to be associated with improved outcomes. The 5-year OS for patients receiving no consolidation, 1 course of consolidation, and ≥2 courses of consolidation were 63.6%, 90%, and 85%, respectively (*p* = 0.0213). The 5-year LFS were 60.6%, 85%, and 60%, respectively (*p* = 0.0561). The 5-year CIR were 9.1%, 7.5%, and 25%, respectively (*p* = 0.3192), and the 5-year NRM were 30.3%, 7.5%, and 15%, respectively (*p* = 0.0426).

## 4. Discussion

Our study demonstrated that pre-transplant consolidation therapy had no significant impact on transplant outcomes in patients with CR2 AML. This is particularly relevant in addressing the dilemma of whether patients should proceed with immediate HSCT in MRD+ status or pursue MRD− through additional consolidation therapy.

The benefit of consolidation chemotherapy in AML patients in CR is to decrease leukemia burden to achieve long-term leukemic control, which has been demonstrated in the non-transplant setting [12,13,14,15]. Thus, the presumed benefit of pre-transplant consolidation chemotherapy for CR2 AML patients is to further reduce the leukemia burden before HSCT for lower relapse rates after transplantation. Some previous studies supported the conclusion that pre-transplant consolidation chemotherapy improves HSCT outcomes in CR1 AML patients [20,21]. However, our data showed no significant difference in transplant outcomes between CR2 AML patients who received consolidation chemotherapy before HSCT and those who did not. Furthermore, consistent with the findings reported by Ciftciler et al. [36], we found that the number of consolidation chemotherapies had no relationship to overall transplantation outcomes.

It is controversial whether multiple courses of chemotherapy increase the risk of treatment-related deaths [37,38,39]. Our study also indicated that age, rather than additional consolidation treatments, was the only independent risk factor associated with an increased risk of NRM, especially considering that 15 patients experienced transplant delays due to infections and other comorbidities.

Although pre-transplant MRD positivity is widely recognized as a strong predictor of post-transplant relapse in AML patients, its prognostic significance is modulated by disease status and transplant timing [40,41,42,43]. Data from non-transplant cohorts have shown that persistent MRD or early relapse following CR1 is associated with poor long-term survival [44]. MRD negativity prior to allo-HSCT is associated with significantly improved outcomes, particularly in patients undergoing transplantation in CR2. A large-scale study by the Acute Leukemia Working Party of the EBMT demonstrated that MRD-negative patients in CR2 had markedly superior LFS and lower CIR compared to their MRD-positive counterparts, highlighting the critical prognostic value of MRD clearance before HSCT [45]. Similarly, a study reported the highest prognostic value with very dismal outcomes for patients who were MRD+ when they received transplants in CR2 [46]. A multicenter retrospective analysis revealed that shorter CR1 duration and elevated HCT-CI were independently linked to worse post-HSCT outcomes in CR2 patients, suggesting that both disease biology and patient fitness should be integrated with MRD status when assessing transplant eligibility and timing [47]. Meanwhile, studies have demonstrated that haplo-HSCT offers a superior GVL effect compared to MSDT in eliminating positive MRD [25,48]. In this study, LFS and OS were comparable between MRD-positive patients undergoing immediate HSCT and MRD-negative patients receiving consolidation. However, this should be interpreted cautiously, as CIR was significantly higher in the MRD-positive group. The lack of difference in survival may be partly due to a trend toward higher NRM in the MRD-negative group, possibly related to treatment delays and complications in the consolidation cohort. Additionally, optimizing the conditioning regimen for CR2 MRD+ patients to reduce tumor burden presents a promising approach in the future. Recent studies on Mega-Dose decitabine have shown significant efficacy in reducing relapse rates in NR-AML [49], suggesting its potential for application in CR2 MRD+ populations.

This study had several limitations. It was a single-center retrospective study, and there were inherent selection biases and baseline imbalances between the consolidation and non-consolidation groups. Our cohort only included patients who successfully underwent allo-HSCT, while data on patients who received consolidation therapy but subsequently failed to proceed to transplant—due to relapse, toxicity, or other complications—were not available. This limitation arises from the fact that a substantial number of transplant recipients were referred to our center from external leukemia services after achieving CR2. As a result, our analysis may underestimate the potential risks of consolidation therapy and does not fully capture its impact on overall transplant eligibility. Second, the number of patients with pre-transplant MRD-positive status is insufficient, which may have affected the results across different subgroups of pre-HSCT courses of consolidation chemotherapy. The relatively small sample size within each risk subgroup also limited our ability to conduct meaningful subgroup analyses based on specific genetic alterations. Future studies with larger, molecularly annotated cohorts are warranted to explore the prognostic implications of specific genetic features in AML patients undergoing HSCT in CR2. Another limitation of this study is the predominance of haplo-HSCT, which accounted for over 90% of all transplants in our cohort; they may have limited generalizability to countries where matched sibling or unrelated donor transplants are more prevalent. Therefore, a prospective study involving a consecutive cohort of CR2 patients—regardless of whether they ultimately proceed to transplant—is warranted to further validate our findings.

## 5. Conclusions

In conclusion, pre-transplant consolidation chemotherapy does not appear to confer additional benefit to AML patients undergoing HSCT in CR2. Therefore, we recommend that all AML patients achieving CR2 after relapse proceed directly to HSCT, irrespective of MRD status, if a suitable donor is available.

## Figures and Tables

**Table 1:** Characteristics of patients.

Characteristics	Total (*n* = 135)	Non-Consolidation Group (*n* = 63)	Consolidation Group (*n* = 72)	*p* Value
Age, years, mean (SD)	30.5 (13.6)	31.0 (15.3)	30.0 (12.1)	0.6668
Sex, *n* (%)				0.5763
Male	78 (57.8)	38 (60.3)	40 (55.6)	
Female	57 (42.2)	25 (39.7)	32 (44.4)	
AML types, *n* (%)				0.2122
De novo AML	132 (97.8)	60 (95.2)	72 (100)	
Secondary AML	2 (1.5)	2 (3.2)	0 (0)	
Unknown	1 (0.7)	1 (1.6)		
Cytogenetics, *n* (%)				0.1117
Favorable	25 (18.5)	8 (12.7)	17 (23.6)	
Intermediate	90 (66.7)	45 (71.4)	45 (62.5)	
Adverse	12 (8.9)	8 (12.7)	4 (5.6)	
Unknown	8 (5.9)	2 (3.2)	6 (8.3)	
ABO matched grafts, *n* (%)				0.0427
Matched	71 (52.6)	39 (61.9)	32 (44.4)	
Mismatched	64 (47.4)	24 (38.1)	40 (55.6)	
D-R sex, *n* (%)				0.8823
F-M	25 (18.5)	12 (19.0)	13 (18.1)	
Others	110 (81.5)	51 (81.0)	59 (81.9)	
Transplant type, *n* (%)				0.0801
MSDT	8 (5.9)	1 (1.6)	7 (9.7)	
MUDT	1 (0.7)	1 (1.6)	0 (0)	
Haplo-HSCT	126 (93.3)	61 (96.8)	65 (90.3)	
Number of induction cycles from relapse to CR2, *n* (%)				0.0906
1	98 (72.6)	40 (63.5)	58 (80.6)	
2	26 (19.3)	15 (23.8)	11 (15.3)	
>2	10 (7.4)	7 (11.1)	3 (4.2)	
Unknown	1 (0.7)	1 (1.6)		
Pre-transplant MRD, *n* (%)				0.0004
Positive	40 (29.6)	28 (44.4)	12 (16.7)	
Negative	95 (70.4)	35 (55.6)	60 (83.3)	
WBC at diagnosis, ×10^9^/L, median (IQR)	19.1 (8.1–53.6)	17.5 (8.0–49.4)	21.4 (8.2–58.6)	0.5117
Infused MNC, ×10^8^/kg, median (IQR)	8.6 (7.5–9.4)	8.7 (7.5–9.8)	8.6 (7.6–9.3)	0.6172
Infused CD34 cells, ×10^6^/kg, median (IQR)	2.5 (1.7–3.5)	2.7 (1.7–3.6)	2.4 (1.7–3.4)	0.4973
Time from diagnosis to HSCT, months, median (IQR)	18.2 (11.6–25.9)	17.0 (8.9–23.7)	19.5 (12.9–27.3)	0.04315
Follow up after HSCT, months, median (IQR)	55.0 (34.9–72.6)	47.5 (9.9–68.1)	55.9 (40.2–75.4)	0.1468

Abbreviations: FAB, French American British AML classification; D-R sex, donor-recipient gender; F-M, female to male; MSDT, HLA-matched sibling transplantation; MUDT, HLA-matched unrelated donor transplantation; Haplo-HSCT, Haploidentical hematopoietic stem cell transplantation; CR2, second complete remission; MRD, minimal residual disease; WBC, white blood cell; MNC, mononuclear cell; HSCT, allogeneic hematopoietic stem cell transplantation; SD, standard deviations; IQR, interquartile range.

**Table 2:** Effect of pre-transplant consolidation therapy on HSCT.

Outcomes	Total (*n* = 135)	Non-Consolidation Group (*n* = 63)	Consolidation Group (*n* = 72)	
	Prob (95% Confidence Interval)	Prob (95% Confidence Interval)	Prob (95% Confidence Interval)	*p* Value
Overall survival				0.0747
1-year	78.5 (71.9–85.8)	73.0 (62.8–84.8)	83.3 (75.2–92.4)	
3-year	77.8 (71.1–85.1)	68.3 (57.7–80.8)	81.9 (73.5–91.3)	
5-year	75.6 (68.6–83.2)	68.3 (57.7–80.8)	81.9 (73.5–91.3)	
Leukemia-free survival				0.1479
1-year	75.6 (68.6–83.2)	69.8 (59.4–82.1)	80.6 (71.9–90.2)	
3-year	73.3 (66.2–81.2)	65.1 (54.3–78.0)	75.0 (65.6–85.7)	
5-year	68.0 (60.3–76.6)	62.7 (51.5–76.2)	72.7 (62.7–84.2)	
Cumulative relapse rate				0.5416
1-year	11.9 (6.4–17.3)	14.3 (5.6–23.0)	9.7 (2.8–16.1)	
3-year	16.3 (10.0–22.6)	17.5 (8.0–26.9)	15.3 (6.9–23.7)	
5-year	18.7 (11.8–25.6)	19.9 (9.5–30.2)	17.6 (8.3–27.0)	
Non-relapse mortality				0.2019
1-year	12.6 (7.0–18.2)	15.9 (6.8–25.0)	9.7 (2.8–16.6)	
3-year	13.3 (7.6–19.1)	17.5 (8.0–26.9)	9.7 (2.8–16.6)	
5-year	13.3 (7.6–19.1)	17.5 (8.0–26.9)	9.7 (2.8–16.6)	
Chronic graft-versus-host disease				0.2872
1-year	43.0 (34.6–51.4)	39.7 (27.4–51.9)	45.8 (34.2–57.5)	
3-year	45.2 (36.7–53.6)	39.7 (27.4–51.9)	50.0 (38.3–61.7)	
5-year	46.4 (37.8–55.0)	39.7 (27.4–51.9)	52.2 (40.2–64.2)	
II–IV aGvHD				0.8436
	18.5 (11.9–25.1)	19.0 (9.3–28.8)	18.1 (9.1–27.0)	

Abbreviations: Prob, probability.

**Table 3:** Multivariable analysis of pre-transplant consolidation therapy with all variables in HSCT setting.

	Multivariable Analysis		
	Hazard Ratio	95% Confidence Interval	*p* Value
Overall survival			
Consolidation vs. Non-consolidation	0.8261	0.3850–1.7722	0.6237
Age (≥30 vs. <30 years)	2.1127	0.9791–4.5584	0.0566
D-R sex (F-M vs. Others)	0.5896	0.2043–1.7014	0.3286
Number of induction cycles from relapse to CR2 (≥2 vs. 1)	1.7439	0.8223–3.6984	0.1471
Pre-transplant MRD (Positive vs. Negative)	1.2713	0.5826–2.7741	0.5466
Time from diagnosis to HSCT (≥18.2 months vs. <18.2 months)	0.4400	0.1998–0.9689	0.0415
Leukemia-free survival			
Consolidation vs. Non-consolidation	0.9647	0.4945–1.8822	0.9162
Age (≥30 vs. <30 years)	1.7776	0.9281–3.4046	0.0827
D-R sex (F-M vs. Others)	0.5003	0.1934–1.2943	0.1533
Number of induction cycles from relapse to CR2 (≥2 vs. 1)	1.9942	1.0212–3.8940	0.0432
Pre-transplant MRD (Positive vs. Negative)	1.5769	0.7813–3.1827	0.2036
Time from diagnosis to HSCT (≥18.2 months vs. <18.2 months)	0.6824	0.3589–1.2974	0.2437
Cumulative relapse incidence			
Consolidation vs. Non-consolidation	2.1625	0.7507–6.2299	0.1531
ABO matched grafts (Mismatched vs. Matched.)	0.5440	0.2092–1.4149	0.2119
D-R sex (F-M vs. Others)	0.2551	0.0332–1.9628	0.1895
Number of induction cycles from relapse to CR2 (≥2 vs. 1)	2.5584	0.9561–6.8461	0.0614
Pre-transplant MRD (Positive vs. Negative)	4.2645	1.4220–12.7892	0.0096
WBC at diagnosis (≥19 × 10^9^/L vs. <19 × 10^9^/L)	0.7220	0.2294–2.2725	0.5777
Non-relapse mortality			
Consolidation vs. Non-consolidation	0.8365	0.2815–2.4861	0.7480
Age (≥30 vs. <30 years)	7.8466	1.4094–43.6856	0.0187
Cytogenetics (Intermediate and Adverse vs. Favorable)	0.9921	0.1714–5.7419	0.9929
ABO matched grafts (Mismatched vs. Matched.)	1.9205	0.6923–5.3278	0.2100
Number of induction cycles from relapse to CR2 (≥2 vs. 1)	1.5460	0.4968–4.8111	0.4520
Time from diagnosis to HSCT (≥18.2 months vs. <18.2 months)	0.4189	0.1296–1.3544	0.1462
Chronic graft-versus-host disease			
Consolidation vs. Non-consolidation	1.1889	0.6619–2.1357	0.5625
Sex (Male vs. Female)	1.3444	0.7661–2.3594	0.3024
ABO matched grafts (Mismatched vs. Matched.)	0.9071	0.5305–1.5511	0.7217
Number of induction cycles from relapse to CR2 (≥2 vs. 1)	0.6981	0.3553–1.3717	0.2970
WBC at diagnosis (≥19 × 10^9^/L vs. <19 × 10^9^/L)	1.5921	0.9084–2.7904	0.1043
Time from diagnosis to HSCT (≥18.2 months vs. <18.2 months)	1.3111	0.7558–2.2746	0.3352
Grade II–IV acute graft-versus-host disease			
Consolidation vs. Non-consolidation	0.7256	0.3072–1.7139	0.4646
Age (≥30 vs. <30 years)	0.5040	0.2010–1.2636	0.1440
Sex (Male vs. Female)	0.5744	0.2329–1.4168	0.2287
Cytogenetics (Intermediate and Adverse vs. Favorable)	0.4797	0.1925–1.1952	0.1147
ABO matched grafts (Mismatched vs. Matched.)	2.2477	0.8916–5.6665	0.0860
D-R sex (F-M vs. Others)	0.5447	0.1271–2.3334	0.4131

The first six variables with the smallest *p* values in the univariable analysis were entered into a multivariable model.

## Data Availability

Data will be made available on request to corresponding author.

## Supplementary Materials

The following supporting information can be downloaded at: https://www.mdpi.com/article/10.3390/cancers17081364/s1, Figure S1: Pre-HSCT MRD level; Table S1: Reason for additional consolidation; Table S2: Pre-HSCT MRD level; Table S3: Univariable analysis of pre-transplant consolidation therapy with all variables in the HSCT setting; Table S4: Effect of the number of pre-transplant consolidation therapy courses on HSCT outcomes.
