# Supplementary material for: Lack of Evidence Supporting a Significant Benefit of Pre-Transplant Consolidation Therapy in AML CR2 Patients Undergoing Allogeneic Hematopoietic Stem Cell Transplantation
Source: Cancers (Basel). 2025 Apr 19;17(8):1364. doi: 10.3390/cancers17081364 (PMC12025703; doi:10.3390/cancers17081364)
Supplement: Supplementary file 1 [file cancers-17-01364-s001.zip › cancers-3518850-supplementary.pdf]

Supplement Table S1 Reason for additional consolidation

n (%)		consolidation group (n=72)	1 consolidation group (n=50)	≥2 consolidation group (n=22)
Preseasons for additional Consolidation	Intention to reduce MRD	50(69.4)	33 (66.0)	17 (77.3)
	Infection or other commodities	15(20.8)	12 (24.0)	3 (13.6)
	Waiting for available donor	7 (9.7)	5 (10.0)	2 (9.1)

Supplement Table S2 Pre-HSCT MRD level

n (%)		Non-consolidation group (n=63)	consolidation group (n=72)	P value
Pre-HSCT MRD Level	<0.01%	35 (55.6)	60(83.3)	0.003
	0.01-1%	15 (23.8)	9 (12.5)	
	>1%	13 (20.6)	3 (4.3)	

n (%)		1 consolidation group (n=50)	≥2 consolidation group (n=22)	P value
Pre-HSCT MRD Level	<0.01%	37 (74.0)	20 (90.9)	0.1719
	0.01-1%	7 (14.0)	2 (9.1)	
	>1%	3 (6.0)	0 (0)	

Supplement Figure S1 Pre-HSCT MRD level

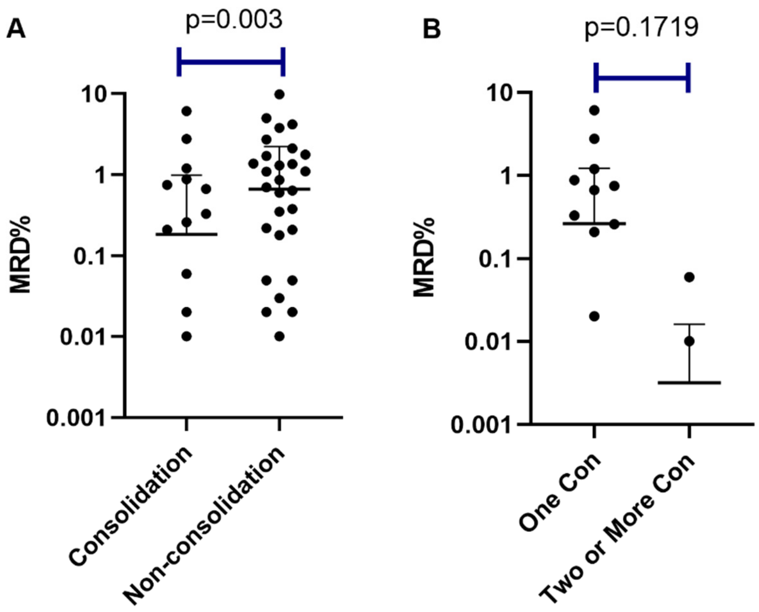

Supplement Table S3

Univariable analysis of pre-transplant consolidation therapy with all variables in the HSCT setting.

	Univariable analysis		
	Hazard ratio	95% confidence interval	P value
Overall survival			
Consolidation vs. Non-consolidation	0.5353	0.2662-1.0766	0.0747
Age (⩾30 vs. <30 years)	2.1720	1.0529-4.4805	0.0313
Sex (Male vs. Female)	0.8817	0.4443-1.7495	0.7176
Cytogenetics (Intermediate & Adverse vs. Favorable)	1.2283	0.4716-3.1991	0.6727
FAB type (M1,2,4,5 vs. any other)	1.2873	0.1759-9.4215	0.8034
ABO matched grafts (Mismatched vs Matched.)	1.2567	0.6349-2.4874	0.5108
D-R sex (F-M vs. Others)	0.5879	0.2067-1.6723	0.3131
Transplant type (MSDT & MUDT vs. Haplo-HSCT)	0.3887	0.0531-2.8451	0.3344
Number of induction cycles from relapse to CR2 (⩾2 vs. 1)	2.2996	1.1423-4.6295	0.0165
Pre-transplant MRD (Positive vs. Negative)	1.4942	0.7252-3.0789	0.2733
WBC at diagnosis (⩾19×10^9/L vs. <19×10^9/L)	0.7988	0.3937-1.6206	0.5315
Time from diagnosis to HSCT (⩾18.2 months vs. <18.2 months)	0.3739	0.1779-0.7859	0.0069
Leukemia-free survival			
Consolidation vs. Non-consolidation	0.6435	0.3524-1.1749	0.1479
Age (⩾30 vs. <30 years)	1.8641	1.0036-3.4626	0.0451
Sex (Male vs. Female)	1.0112	0.5517-1.8536	0.9721
Cytogenetics (Intermediate & Adverse vs. Favorable)	1.0015	0.4414-2.2721	0.9978
FAB type (M1,2,4,5 vs. any other)	0.5127	0.1583-1.6599	0.2559
ABO matched grafts (Mismatched vs Matched.)	0.9991	0.5486-1.8196	0.9976
D-R sex (F-M vs. Others)	0.5343	0.2102-1.3579	0.1803
Transplant type (MSDT & MUDT vs. Haplo-HSCT)	0.3183	0.0437-2.3157	0.2327
Number of induction cycles from relapse to CR2 (⩾2 vs. 1)	2.1617	1.1658-4.0085	0.0122
Pre-transplant MRD (Positive vs. Negative)	1.4737	0.7800-2.7844	0.2289
WBC at diagnosis (⩾19×10^9/L vs. <19×10^9/L)	0.7511	0.3988-1.4145	0.3729
Time from diagnosis to HSCT (⩾18.2 months vs. <18.2 months)	0.5851	0.3190-1.0730	0.0796
Cumulative relapse incidence			
Consolidation vs. Non-consolidation	0.7831	0.3594-1.7060	0.5416
Age (⩾30 vs. <30 years)	0.9090	0.4182-1.9757	0.8166
Sex (Male vs. Female)	0.9281	0.4237-2.0328	0.8556
Cytogenetics (Intermediate & Adverse vs. Favorable)	0.7809	0.2889-2.1111	0.6171
FAB type (M1,2,4,5 vs. any other)	0.4357	0.1133-1.6757	0.2739
ABO matched grafts (Mismatched vs Matched.)	0.5930	0.2628-1.3378	0.2025

D-R sex (F-M vs. Others)	0.3514	0.0843-1.4643	0.1295
Transplant type (MSDT & MUDT vs. Haplo-HSCT)	0.6335	0.0864-4.6443	0.6490
Number of induction cycles from relapse to CR2 (≥2 vs. 1)	2.0168	0.9068-4.4853	0.0864
Pre-transplant MRD (Positive vs. Negative)	3.5609	1.5771-8.0402	0.0019
WBC at diagnosis (≥19×10^{9}/L vs. <19×10^{9}/L)	0.4773	0.1928-1.1814	0.1042
Time from diagnosis to HSCT (≥18.2 months vs. <18.2 months)	0.8342	0.3845-1.8096	0.6469
Non-relapse mortality			
Consolidation vs. Non-consolidation	0.5444	0.2116-1.4001	0.2019
Age (≥30 vs. <30 years)	5.3250	1.5301-18.5314	0.0031
Sex (Male vs. Female)	1.1533	0.4499-2.9562	0.7678
Cytogenetics (Intermediate & Adverse vs. Favorable)	1.6973	0.3721-7.7409	0.4802
FAB type (M1,2,4,5 vs. any other)	0.6662	0.1004-4.4192	0.6893
ABO matched grafts (Mismatched vs Matched.)	1.8729	0.7358-4.7673	0.1881
D-R sex (F-M vs. Others)	0.8926	0.2558-3.1143	0.8579
Number of induction cycles from relapse to CR2 (≥2 vs. 1)	1.9174	0.7425-4.9512	0.1800
WBC at diagnosis (≥19×10^{9}/L vs. <19×10^{9}/L)	1.2512	0.4989-3.1380	0.6360
Time from diagnosis to HSCT (≥18.2 months vs. <18.2 months)	0.4566	0.1740-1.1981	0.1081
Chronic graft-versus-host disease			
Consolidation vs. Non-consolidation	1.3164	0.7912-2.1901	0.2872
Age (≥30 vs. <30 years)	1.1338	0.6912-1.8598	0.6161
Sex (Male vs. Female)	1.1489	0.6967-1.8947	0.5895
Cytogenetics (Intermediate & Adverse vs. Favorable)	0.8497	0.4377-1.6495	0.6089
FAB type (M1,2,4,5 vs. any other)	1.3445	0.3734-4.8414	0.6658
ABO matched grafts (Mismatched vs Matched.)	0.8329	0.5083-1.3649	0.4695
D-R sex (F-M vs. Others)	0.9154	0.4825-1.7367	0.7855
Transplant type (MSDT & MUDT vs. Haplo-HSCT)	0.8685	0.3474-2.1711	0.7828
Number of induction cycles from relapse to CR2 (≥2 vs. 1)	0.6530	0.3542-1.2038	0.1622
Pre-transplant MRD (Positive vs. Negative)	0.9735	0.5644-1.6791	0.9187
WBC at diagnosis (≥19×10^{9}/L vs. <19×10^{9}/L)	1.7102	1.0033-2.9152	0.0501
Time from diagnosis to HSCT (≥18.2 months vs. <18.2 months)	1.3493	0.8189-2.2234	0.2418
Grade II-IV acute graft-versus-host disease			
Consolidation vs. Non-consolidation	0.9241	0.4240-2.0142	0.8436
Age (≥30 vs. <30 years)	0.5288	0.2344-1.1930	0.1200
Sex (Male vs. Female)	0.6546	0.3005-1.4258	0.2871
Cytogenetics (Intermediate & Adverse vs. Favorable)	0.4429	0.1931-1.0161	0.0588
ABO matched grafts (Mismatched vs Matched.)	2.1125	0.9395-4.7499	0.0666
D-R sex (F-M vs. Others)	0.5651	0.1730-1.8463	0.3392

Number of induction cycles from relapse to CR2 (≥2 vs. 1)	1.3578	0.5852-3.1504	0.4786
Pre-transplant MRD (Positive vs. Negative)	0.7949	0.3145- 2.0086	0.6251
WBC at diagnosis (≥19×10^9/L vs. <19×10^9/L)	1.1965	0.5221-2.7419	0.6744
Time from diagnosis to HSCT (≥18.2 months vs. <18.2 months)	0.7490	0.3424-1.6384	0.4713

Supplement Table S4

Effect of the number of pre-transplant consolidation therapy courses on HSCT outcomes.

Outcomes	Non-consolidation group (n=63) Prob (95% confidence interval)	1 consolidation group (n=50) Prob (95% confidence interval)	≥2 consolidation group (n=22) Prob (95% confidence interval)	P value
Overall survival				0.2043
1-year	73.0 (62.8-84.8)	84.0 (74.4-94.8)	81.8 (67.2-99.6)	
3-year	68.3 (57.7-80.8)	82.0 (72.0-93.4)	81.8 (67.2-99.6)	
5-year	68.3 (57.7-80.8)	82.0 (72.0-93.4)	81.8 (67.2-99.6)	
Leukemia-free survival				0.1973
1-year	69.8 (59.4-82.1)	84.0 (74.4-94.8)	72.7 (56.3-93.9)	
3-year	65.1 (54.3-78.0)	78.0 (67.3-90.4)	68.2 (51.3-90.7)	
5-year	62.7 (51.5-76.2)	78.0 (67.3-90.4)	59.7 (40.5-87.9)	
Cumulative relapse rate				0.5855
1-year	14.3 (5.6-23.0)	8.0 (0.4-15.6)	13.6 (0-28.4)	
3-year	17.5 (8.0-26.9)	14.0 (4.3-23.7)	18.2 (1.6-34.8)	
5-year	19.9 (9.5-30.2)	14.0 (4.3-23.7)	26.7 (4.3-49.1)	
Non-relapse mortality				0.3631
1-year	15.9 (6.8-25.0)	8.0 (0.4-15.6)	13.6 (0-28.4)	
3-year	17.5 (8.0-26.9)	8.0 (0.4-15.6)	13.6 (0-28.4)	
5-year	17.5 (8.0-26.9)	8.0 (0.4-15.6)	13.6 (0-28.4)	
Chronic graft-versus-host disease				0.4727
1-year	39.7 (27.4-51.9)	46.0 (32.0-60.0)	45.5 (23.9-67.1)	
3-year	39.7 (27.4-51.9)	48.0 (33.9-62.1)	54.5 (32.7-76.3)	
5-year	39.7 (27.4-51.9)	48.0 (33.9-62.1)	63.6 (38.3-89.0)	
Grade II-IV acute graft-versus-host disease				0.3541
100-day	19.0 (9.3-28.8)	14.0 (4.3-23.7)	27.3 (8.2-46.4)	

Abbreviations: Prob, probability.
